# Mutual relationships between SARS-CoV-2 test numbers, fatality and morbidity rates

**DOI:** 10.1186/s12889-021-12021-y

**Published:** 2021-11-02

**Authors:** Piotr Korneta, Janusz Zawiła-Niedźwiecki, Jarosław Domański

**Affiliations:** Faculty of Management, Warsaw University of Technology, ul. Narbutta 85, 02-524 Warszawa, Poland

**Keywords:** SARS-CoV-2, Testing strategy, Pandemic, Power law, Probabilistic approach, Multi Theil Sen

## Abstract

**Background:**

The number of SARS-CoV-2 tests conversely to other factors, such as age of population or comorbidities, influencing SARS-CoV-2 morbidity and fatality rates, can be increased or decreased by decision makers depending on the development of the pandemic, operational capacity, and financial restraints. The key objective of this study is to identify and describe, within the probabilistic approach, the relationships between SARS-CoV-2 test numbers and the mortality and morbidity rates.

**Methods:**

The study is based on a statistical analysis of 1058 monthly observations relating to 107 countries, from six different continents, in an 11-month period from March 2020 to January 2021. The variable utilised can be defined as the number of tests performed in a given country in 1 month, to the number of cases reported in a prior month and morbidities and mortalities per 1 million population. The probabilities of different mortality and morbidity rates for different test numbers were determined by moving percentiles and fitted by the power law and by the three-segment piecewise-linear approximation based on Theil Sen trend lines.

**Results:**

We have identified that for a given probability the dependence of mortality and morbidity rates on SARS-CoV-2 test rates follows a power law and it is well approximated by the three Theil Sen trend lines in the three test rate ranges. In all these ranges Spearman rho and Kendall tau-b rank correlation coefficients of test numbers and morbidity with fatality rates have values between − 0.5 and − 0.12 with *p*-values below 0.002.

**Conclusions:**

According to the ABC classification: the most important, moderately important, and relatively unimportant ranges of test numbers for managing and control have been indicated based on the value of the Theil Sen trend line slope in the three SARS-CoV-2 test rate ranges identified. Recommendations for SARS-CoV-2 testing strategy are provided.

## Background

The SARS-CoV-2 pandemic has given rise to many difficult challenges for political and social decision makers. This is primarily because governments should not only be effective in terms of protecting the life and health of their citizens, but should also remain organisationally rational (e.g., where to act in the first place and how to act) and cost-efficient. The latter raises the need to find scientific tools to measure the intensity of the pandemic phenomenon and its dynamics over time, in the human population and in the area administered. It is also particularly important to be able to measure the effectiveness of pandemic limitation processes and the results of actions undertaken by governments.

Pursuant to the onset of the pandemic, despite the relatively short period, many papers on SARS-CoV-2 appeared. These papers, however, focus primarily on the medical aspects of SARS-CoV-2, biochemistry, immunology, and microbiology. It has been acknowledged that the infection can develop within utterly different scenarios. A fraction of SARS-CoV-2 patients may develop mild symptoms, which do not require any treatment; while another fraction might produce severe respiratory distress, requiring hospitalisation in intensive care units [1–4]. The latter group requires considerably more attention from clinicians and scientists. The world fatality rate for SARS-CoV-2 is estimated to be around 4% of infected patients, varying from 0 to 20% among different countries [3, 5, 6]. The key challenge while measuring fatality rates appears to be the denominator, as not all infections might be diagnosed, resulting in overestimations of mortality rates [5, 7]. A significant body of literature has already confirmed age, obesity, and comorbidities, including, inter alia, diabetes, cardiovascular diseases, and cancers increase the SARS-CoV-2 fatality risk [8–11]. Considerable research has also been done relating to laboratory variables indicating higher fatality rates for patients with already diagnosed SARS-CoV-2 [6]. There is also some research relating to associated factors with higher mortality rates, including demographic, economic, and political variables as well as fatality rates among various countries [12]. Many countries measure the development of the pandemic with the reproduction number of an infectious disease denoted by R, which is defined as the average number of secondary cases produced by a primary case [13, 14]. Values of R below 1 indicate the number of infections decreases, while the values above 1 indicate the opposite, i.e., the expansion of the pandemic. As a result, the ratio plays a significant role in public policy decisions, e.g., assessing the effectiveness of non-pharmaceutical interventions has already been subject to many studies [15–17]. The reproduction number R varies over time and is influenced by many factors, e.g., duration of infection, transmission possibilities, susceptibility (i.e., older people are more likely to get infected and to develop a severe form of infection), country testing strategy, implementation of lock downs, and others. Its value is therefore determined with great error. Despite, as indicated, the already developed SARS-CoV-2 literature, little has been done in relation to quantities, over which decision makers have control, and which can be efficiently employed against the pandemic. Among the areas which require further studies are SARS-CoV-2 testing strategies, excluding, however, studies on test sensitivity and accuracy, as we consider that a significant job in this area has already been done [18, 19]. Tests are widely acknowledged to limit the spread of the pandemic through identification of major sources of transmission [20, 21]. Despite what many scholars postulate, to increase the number of tests performed, which should limit further expansion of the pandemic [22], there are only a few empirical studies that provide further insight on that matter. Some scholars indicate the virus might have spread faster in poorer regions due to testing limitations resulting from high costs [23], and that the number of tests performed relates more to the country’s financial status than the extent of pandemic development in a particular country [24]. The cost aspect seems to play a significant role, as global healthcare systems have already incurred considerable expenses related to SARS-CoV-2 treatment, with some countries considering painful cost rationings [25]. The initial researchers have previously confirmed negative associations between the number of tests and SARS-CoV-2 mortality on small statistical samples, during the early stages of the pandemic [7]. To the best of our knowledge, no researchers thus far have provided optimum test levels and neither the influence of different test ranges on the development of the pandemic nor the probabilities of morbidity and fatality rates.

Consequently, the objective of this paper is to identify and measure the relationships between the number of SARS-CoV-2 tests and the probabilities of SARS-CoV-2 morbidity and fatality rates. We assume that different ranges of anti-SARS-CoV-2 tests may have different associations with the probabilities of both the fatality and morbidity rates. In our study, we aim to identify and analyse these ranges. To the best of our knowledge, this study is the first to explore the relationships between different ranges of SARS-CoV-2 tests and the probabilities of morbidity and fatality rates.

In this paper, we analysed data relating to 107 countries from six continents in an 11-month period from March 2020 to January 2021. We applied a probabilistic approach which considers that randomness plays a role in predicting the spread of epidemics. This approach gives only probabilities of possible outcomes and provides a more complete picture of future events than it is possible to derive from a deterministic analysis. The probabilistic approach resolves the problem posed by the limits of historical data used in this paper. Because of significant data dispersion, we had to use robust statistical methods in our analysis. We applied moving median and percentiles to determine the form of the relationship between the number of SARS-CoV-2 tests and the selected probabilities of mortality and morbidity of SARS-CoV-2 rates in their different ranges. Spearman rho and Kendall tau-b [26, 27] were used to determine the significance of the correlations in each range. The power law and the multi-segment piecewise-linear approximation based on Theil Sen trend lines [28] have been used to fit the relations obtained. Our analysis allowed us to formulate recommendations for dealing with the pandemic on how to improve their SARS-CoV-2 testing strategies. State decision makers are the first to directly benefit from the results obtained in this study. Of note, we focus on mutual relationships between the test numbers and morbidity and fatality rates, with limited presentation and explanation of the diagnostic process.

The paper is organised as follows: in the following section, research methods and their different components such as the database and sample of observations, variables, and design of the study are given. The next section presents the results derived from the empirical data. The following section provides discussion and the limitations of our study. Finally, we give conclusions and implications for various groups of stakeholders.

## Methods

### Database and sample of observations

The data used in this study were retrieved from the open access database “Our World in Data” [29]. This database has compiled official information relating to polymerase chain-reaction (PCR) testing around the World [30] and has already been used in other studies on the SARS-CoV-2 pandemic [31]. We obtained from this database, as of 7 February 2021, 64,360 daily observations relating to 107 countries in an 11-month period from March 2020 to January 2021. Pursuant to a data reliability evaluation, which mostly comprised analysis of outliers and missing observations, we moved further and aggregated the daily observations obtained into 1058 monthly observations. As a result, we studied figures relating to 1.0806 billion anti-SARS-CoV-2 tests undertaken, 82.5 million new cases reported, and 1.7 million SARS-CoV-2 deaths. Following completion of each step of the research which required data processing, we verified the data reliability, ensuring, inter alia, the sum of the total had not changed. In Table 1, we provide the breakdown of a sample of observations used in the study divided by continent and month of the period under investigation.

**Table 1:** The sample of observations used in the study

	Number of monthly observations (Mar 2020 – Jan 2021)												Number of countries
Continent/month	3	4	5	6	7	8	9	10	11	12	1	Total	
Africa	0	19	21	22	22	22	23	22	21	20	19	211	**23**
Asia	12	25	25	26	27	28	27	28	28	28	28	282	**28**
Europe	17	31	33	33	34	32	34	34	34	34	34	350	**34**
North America	3	10	11	11	11	11	11	11	10	10	9	108	**11**
Australia&Oceania	1	3	3	2	2	3	3	3	3	3	3	29	**3**
South America	0	7	7	8	8	8	8	8	8	8	8	78	**8**
Total	**33**	95	100	102	104	104	106	106	104	103	101	1058	**107**

### Variables

The average SARS-CoV-2 incubation period is 4–7 days, the time interval between symptom onset and hospital admission is around 3–10 days, and the time interval between hospital admission and death is between 5 to 16 days [32]. Given the time of these intervals, the approximated time from infection to death is around a month. As a result, in our study we employed monthly observations to capture the average time between the infection and the reported case. Let us denote by *C*_*i*_ and *D*_*i*_ the number of infected and dead individuals reported in a country in the *i*-th month. We define the SARS-CoV-2 case rate (CCR) and the covid fatality rate (CFR) normalised for per million of population as follows: *CCR* = 10^6^*C*_*i*_/*P* and *CFR* = 10^6^*D*_*i*_/*P*, where *P* denotes the country’s population. These variables have already been employed by other scholars [24]. We also defined the efficiency of testing as quantified by the covid test rate (CTR) defined as: *CTR* = *T*_*i*_/*C*_*i* − 1_, were *T*_*i*_ is the number of tests reported in a country in the *i*-th month and *C*_*i* − 1_ is the number of new SARS-CoV-2 cases diagnosed in the preceding month. We calculated variables CCR, CFR and CTR for each of the months and for each country studied, resulting in a set of observations. The analysis of these observations allows us to assess the probability of different morbidity and mortality rates in the next month based on the number of SARS-CoV-2 cases reported in a given month, assuming a different number of planned tests. The acronyms and description of the variables used in this paper are provided in Table 2.

**Table 2:** The variables used in this study

Acronym	Variable	Description
CCR	SARS-CoV-2 cases rate	New SARS-CoV-2 cases reported in a month to one million of population of the country
CFR	SARS-CoV-2 fatality rate	New SARS-CoV-2 deaths reported in a month to one million of population of the country
CTR	SARS-CoV-2 tests rate	Number of SARS-CoV-2 tests performed in a country in a month to the number of SARS-CoV-2 cases reported in a prior month

### Design and data analysis

Once the variables had been selected, we analysed the data using descriptive statistics and visualised the relationships between the variables studied in graphs. The visualisation of these relationships was achieved using a moving median to determine the different relations between the variables in their different ranges. We calculated the median values of the two variables considered in a moving window containing data of 50 and 200 observations. The moving median is a robust measure of the central tendency in the sliding window content, while the moving average is not (it is overly sensitive to strong outliers and the non-Gaussian distribution of data in a window) [33]. As a result, we obtained a curve by dividing the local data in the window in two. We successfully fitted this curve using the power law. The power law is a functional relationship between two quantities, where one quantity varies as a power of another [34]. A considerable variety of, inter alia, biological, medical, economical, and physical quantities follow, or approximately follow, a power law [35–37]. The power law distribution (also known as a Pareto distribution) for a particular set of parameters describes the 80/20 rule (called the Pareto principle) which states that 80% of the consequences come from 20% of the causes. In practice this proportion may be different. This principle allows priorities to be set and maximum results to be achieved. It has been suggested to use it to explain transmission events during epidemics [38]. Closely associated with the Pareto principle is an ABC classification. This classification has become a popular business metrics, especially for inventory management [39, 40]. The division of our relations into 3 segments, namely: A - the most important, B - moderately important, and C relatively unimportant, identifies ranges of epidemic intensity with differing effects on public health. We calculated the Spearman rho and the Kendall tau-b coefficients in each range. Both statistics are non-parametric rank correlation measures, widely used to measure the ordinal associations between two quantities [26, 27]. Next, we applied the Theil-Sen procedure to the observations in each range and fitted the relation between the variables considered by a straight line. The Theil-Sen procedure is acknowledged to be the most common nonparametric technique for estimating a linear trend [28]. The objective of this method is to fit a line to sample points robustly by choosing the median of the slopes of all lines through pairs of points [32–44]. The Theil–Sen estimator is considerably more accurate than non-robust simple linear regression, especially for skewed and heteroskedastic data. Additionally, it should be noted, the Theil–Sen estimator provides better results than the non-robust least squares method, even for normally distributed data [45]. As a result of this approach, we obtained multi-segment piecewise-linear approximation to the relationships between the variables under investigation. We note that this approach has been already successfully used in other studies [46]. In this paper we applied the probabilistic approach, which generates the probabilities of the possible outcomes, contrary to the deterministic approach giving only a single outcome. We thus calculated percentiles for different probabilities for the relationships between the considered variables in a moving window containing 200 observations. We obtained a set of curves specifying the probabilities of different scenarios. We could fit all these curves for a given relationship between variables by the power law with the same exponent. By multiplying the coefficients of lines, forming a multi-segment piecewise-linear Theil-Sen approximation of the moving median by one factor, we obtained a multi-segment piecewise-linear approximation for each percentile.

Finally, to understand the wider context between the progression of the SARS-CoV-2 pandemic and the number of tests performed, we calculated (in absolute monthly figures) the Spearman correlation and Kendall tau-B for the number of monthly tests performed and monthly numbers of SARS-CoV-2 cases and deaths reported.

## Results

Table 3 shows the descriptive statistics of the variables used in this study calculated for each country in each month over the period studied. There is a high mean number of SARS-CoV-2 case rate, 2432.7, and a low median of 609.2. In half of the studied countries there were up to 609.2 new SARS-CoV-2 cases diagnosed per 1 million population. The maximum of diagnosed SARS-CoV-2 monthly cases (CCR) is 30,092 per 1 million population reported in Portugal, in January 2021. The median of deaths from SARS-CoV-2, per 1 million population, is 8.9 and the maximum is 607 reported in Slovenia, in December 2020. In half of the countries, up to 30.6 more SARS-CoV-2 tests were performed than diagnosed SARS-CoV-2 cases in the previous month. The difference between min. and max. Values, with a high standard deviation, and kurtosis of 344 of CTR variable, indicate the different COVID-19 screening strategies adopted by different countries in different months. The skewness of 17.2 indicates the large asymmetry of the CTR observations, i.e., most observations are below the mean value.

**Table 3:** Descriptive statistics of CCR, CFR and CTR variables

Variable	Mean	SD	Median	Min	Max	Skewness	Kurtosis
CCR	2432.7	4340.9	609.2	0	30,092	3	10.7
CFR	45.7	87	8.9	0	607	3.1	11.2
CTR	506.3	3876.3	30.6	0.1	88,318	17.2	344

**Fig. 1:**
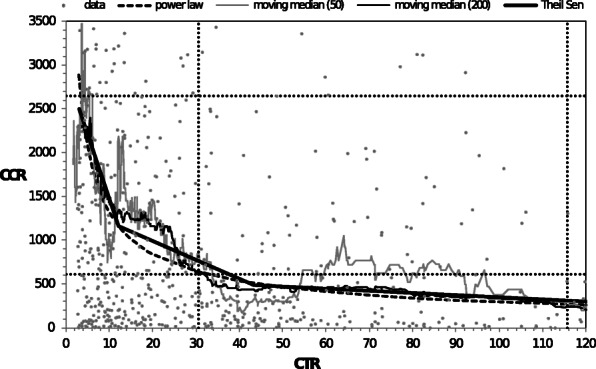
The relationship between CCR and CTR with the moving median from 50 and 200 observations and their power law and three-segment linear Theil Sen approximations. The dotted lines denote positions of the median and the third quartile of CTR and CCR for all data

**Fig. 2:**
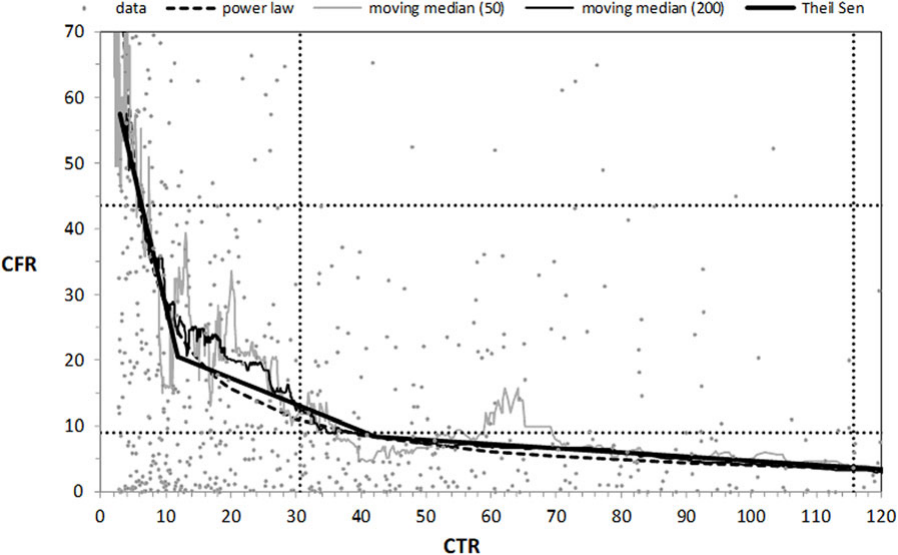
The relationship between CFR and CTR with the moving median from 50 and 200 observations and their power law and three-segment linear Theil Sen approximations. The dotted lines denote the positions of the median and the third quartile of CTR and CFR for all data

In Figs. 1 and 2 we plotted the original data of the relation between CCR, CFR and CTR together with their moving medians calculated in a window ranging from 50 and 200 observations. It can be observed that both moving medians similarly and correctly describe the relationships under investigation. In Figs. 1 and 2 we also show the median and third quartile of CCR and CFR variables for all data (dashed lines). We used the least-squares method to fit the moving median by the following power law functions: *CCR* = 5,900/*CTR*^0.65^ and =200/*CTR*^0.85^ . For the moving median from 200 observations the R-squared measure of the fit quality was above 0.9. The power law curves fitted to the moving median in Figs. 1 and 2 determine approximately for each CTR values of CCR and CFR below which variables CCR and CFR are lower with a probability of 0.5. Because CTR is defined as the number of SARS-CoV-2 tests performed in a given country in a given month, to the number of SARS-CoV-2 cases reported in a prior month, the dependencies shown in Figs. 1 and 2 allow the CCR and CFR values to be predicted. Likewise, in the next month, based on the number of cases reported in each month and assuming a different number of planned tests, this results in a probability of 0.5. The higher exponent of CFR on CTR dependence means that it decreases faster. For a management and controls power law, dependence is usually divided into three ranges identifying which parts of a process are extremely important, moderately important, or relatively unimportant. This is the ABC classification used e.g., for business process management and bankruptcy prediction. We performed the three-segment piecewise-linear Theil-Sen approximation of the relationships considered. The values obtained of Theil-Sen slopes (m) and intercepts (b) are given in Table 4. We identified the three ranges for the following ranges of CTR values: (4–12), (12–43) and greater than 43. These ranges contain 23, 27, and 40% of observations, respectively. The slopes of the Theil-Sen lines in the three ranges differ greatly. For CTR∈(4.12), the Theil-Sen line rapidly decreases with the lowest large coefficient m. The relationships studied in this range are sensitive to any changes in CTR value. This is range A, in the ABC classification, which plays the most important role in epidemic control. In the range of CTR values between 12 and 43, the slope of the Theil-Sen line is considerably lower. This is range B, which requires less attention and control. For CTR > 43, the Theil-Sen line is almost flat, and both CCR and CFR are significantly less sensitive to any changes in CTR values. This is the marginally important range C. In Table 4, we provide the results of the statistical tests of the relationships between CCR, CFR, and CTR in the ranges identified. We show the Spearman rho and the Kendall tau-b values with their *p*-values (one side). These values are negative in all three ranges. Exceptionally low p-values indicate that the results obtained are statistically significant and robust. We performed a linear approximation of the relationships under investigation for observations in the selected ranges using the Theil-Sen procedure. The obtained values of the Theil-Sen slopes (m) and intercepts (b) are given in Table 4. This three-segment piecewise-linear approximation describes the relationships considered in the three identified ranges well. The slopes of the Theil-Sen lines in the three ranges differ greatly. For CTR ∈(4.12), the Theil-Sen line rapidly decreases with the lowest large coefficient m. The relationships in this range are sensitive to any changes in CTR value. This is the range A, in the ABC classification, which plays the most important role in epidemic control. In the range of CTR values between 12 and 43, the slope of the Theil-Sen line is considerably lower. This is range B, which requires less attention and control. For CTR > 43, the Theil-Sen line is almost flat and both CCR and CFR are significantly less sensitive to any changes in CTR values. This is the marginally important range C.

**Table 4:** Results of statistical tests using the Spearman rho and the Kendall tau-b of relations between CCR, CFR and CTR variables with Theil-Sen slopes (m) and intercepts (b) of trend lines approximating these relations in the three ranges

		Spearman	Kendall			Theil-Sen	
Variable	CTR Range	coefficient	p	Tau-B	p	m slope	b intercept
CCR	4–12	−0.146	0.0136	−0.101	0.0115	−146.111	2940.67
CCR	12–43	−0.178	0.0011	−0.123	0.0009	−21.427	1421.19
CCR	> 43	−0.353	< 0.0001	−0.235	< 0.0001	−2.495	599.284
CFR	4–12	−0.203	0.0009	−0.138	0.0009	−4.191	70.092
CFR	12–43	−0.184	0.0007	−0.128	0.0005	−0.398	25.167
CFR	> 43	−0.484	< 0.0001	−0.337	< 0.0001	− 0.064	11.038

As provided in Table 4, the results of the relationships obtained, for each of identified ranges, are negative and statistically significant. Very low *p* values (below 0.0001) in the Spearman coefficient and Kendall tau-b confirm our results are robust.

**Fig. 3:**
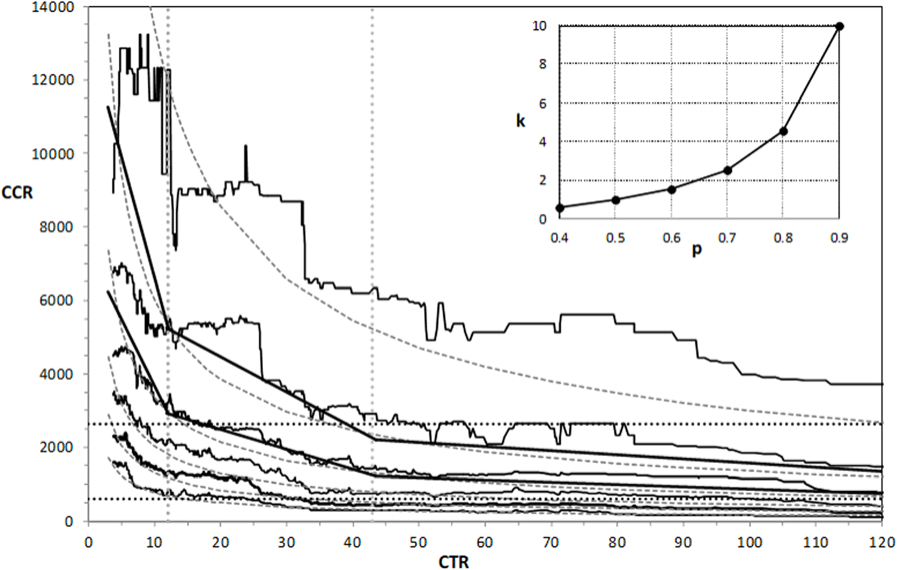
The relationship between CCR and CTR with the moving percentile from 200 observations and their power law approximation =5,900*k*/*CTR*^0.65^ . The percentile lines correspond to probabilities 0.9, 0.8, 0.7, 0.6, 0.5 and 0.4 from top to bottom. The three-segment linear approximations are shown for *p* = 0.8 and *p* = 0.7. The inset shows the dependence of the power law coefficient k on the probability p of percentile. For CTR below grey dotted lines there are 33 and 60% of all data. The black dotted lines denote the positions of the median and the third quartile of CCR for all data

**Fig. 4:**
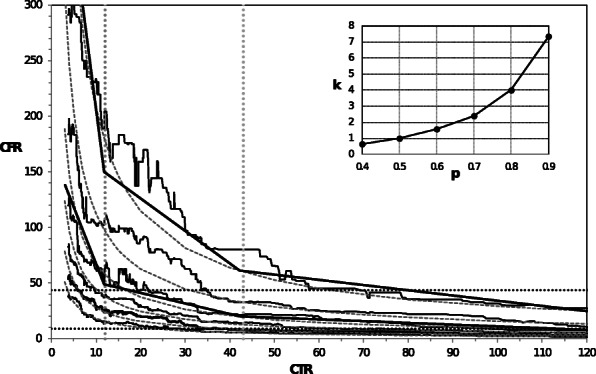
The relationship between CFR and CTR with the moving percentile from 200 observations and their power law approximation =200*k*/*CTR*^0.85^ . The percentile lines correspond to probabilities 0.9, 0.8, 0.7, 0.6, 0.5 and 0.4 from top to bottom. The three-segment linear approximations are shown for *p* = 0.9 and p = 0.7. The inset shows the dependence of the power law coefficient k on the probability p of percentile. For CTR below grey dotted lines, there are 33 and 60% of all data. The black dotted lines denote the positions of the median and the third quartile of CFR for all data

The probabilistic interpretation of the moving-median describing the dependence of CCR and CFR on CTR suggests that the probabilistic approach to the relationships between SARS-CoV-2 test numbers and probabilities of mortality and morbidity rates are based on the moving percentile. In Figs. 3 and 4, we show the dependence of CCR and CFR on CTR with the moving-percentile curves calculated in a window from 200 observations for probabilities between 0.4 and 0.9. These curves were fitted using the least-squares method with R-squared above 0.9 by the following power law functions: *CCR* = 5,900*k*/*CTR*^0.65^ and =200*k*/*CTR*^0.85^ . The worse fit we obtained was only for the dependence of CCR on CTR and *p* > 0.8. The dependence of the coefficient k on the probability p of the percentile is shown in the inset of Figs. 3 and 4. All the moving percentile curves can also be fitted by three-segment piecewise-linear approximation, as shown for selected curves in Figs. 3 and 4. These approximations can be obtained by multiplying the m and b coefficients of the Theil-Sen lines; given in Table 4 by the coefficient k, corresponding to the probability p of a percentile. This shows that the ABC classification and identification of the most important and the marginally important ranges in epidemic control, also occurs for the moving percentile. These relationships are the most sensitive to changes in the test rate in ranges A and B, i.e., up to a CTR value of 43. The dependencies shown in Figs. 3 and 4 allow the values of CCR and CFR in the next month to be predicted based on the number of cases reported in a given month and assuming a different number of planned tests for different probabilities. For CTR = 12, there is e.g., a probability of 0.9 that the new SARS-CoV-2 monthly cases rate will be below 12,000 (persons per 1 million population); a probability of 0.7 that it will be below 3000; and only a probability 0.4 that CCR will be under 700. For CTR = 12, the variable CFR with probabilities 0.9, 0.7 and 0.4 will be below 178, 58, and 16, respectively. For CTR > 43 (C range) the moving percentile curves representing probabilities between 0.4 and 0.7 are close to each other, while the remaining percentile lines representing 0.9 and 0.8 probabilities are just above them. This indicates that the increase in the number of SARS-CoV-2 tests in the C range is inefficient.

Finally, for indicative purposes, we have calculated the Spearman’s rho and the Kendall tau-B for the monthly number of tests performed and the number of Sars-CoV-19 monthly reported cases (Spearman’s rho 0.76; tau-B 0.57) and deaths (Spearman’s rho 0.69; tau-B 0.51). These results indicate strong and positive associations between the number of tests performed and new Sars-CoV-19 cases and deaths. We shall also note that the aforementioned results are robust, with very low *p* values (< 0.0001).

## Discussion

Although a considerable number of factors have already been postulated in the literature to be associated with the SARS-CoV-2 pandemic, little attention has been paid to the factors over which decision makers have control and which can be managed. Factors like, inter alia, obesity, age of population, average life expectancy, and comorbidities [8–12], although they are good indicators for severe or mild pandemic development, are unmanageable, at least in the short term. Among the most critical factors over which decision makers have influence, and which can contribute to pandemic limitation, are SARS-CoV-2 tests. It can be intuitively postulated that it is not possible to limit the pandemic if we do not know who is and who is not infected. Therefore, some academics propose that the number of tests should be increased, which should limit the pandemic’s development [21, 22]. These scholars assume that the significant and negative association between the number of SARS-CoV-2 tests and pandemic proliferation, is measured by the morbidity and fatality rates. The initial results obtained from the first studies claim the existence of negative and statistically significant relationships between the number of tests and pandemic morbidity and fatality rates [24]. The result obtained in our study, performed on a larger sample comprising 107 countries from all 6 continents, for an 11-month period, endorses these results. The relationship between morbidity and fatality rates and the test ratio (CTR), defined here as the number of SARS-CoV-2 tests performed in a given country in a given month, to the number of SARS-CoV-2 cases reported in the preceding month, is in all ranges negative. However, we showed that there is the most important, the moderately important, and relatively unimportant range for managing and control, according to ABC classification. We have econometrically measured the relationship contemplated and approximated it with a power law and a three-segment linear approximation. The numbers of new SARS-CoV-2 cases and deaths reported are sensitive to CTR up to its value of 12. Yet, considerably sensitive up to the CTR value of 43. A further increase in test numbers, i.e., above a CTR value of 43, provides inefficient improvement in the numbers of new SARS-CoV-2 cases or deaths.

Despite the negative association between tests numbers and pandemic development provided above, those countries willing to test large populations face considerable challenges, limiting their testing capabilities. There are several factors limiting the testing capabilities of such countries, among which financial and operational constraints appear to play a critical role. Some scholars postulate that the high costs, or the savings on spending on them, have a considerable impact on pandemic development. These scholars hypothesise that the more affluent countries spend more on tests than the poorer ones. Some of them state that the correlation between a country’s affluence and the number of tests is higher than with medical testing needs [24]. Hence, despite the fact that the protection of human lives and health is of utmost importance, due to the high costs of testing, some countries limit their expenditure. This is also because global healthcare systems have already incurred considerable SARS-CoV-2 treatment costs [25]. The limitation of operational capabilities arises primarily from the throughput of testing machines, i.e., the number of tests completed per testing cycle and the number of testing cycles per day, testing personnel shortages, and the supply of testing materials [47–49]. Given that there is a considerable limitation in implementation of high-volume testing, this paper adds the efficiency of testing in different ranges to the literature and recommends two thresholds, depending on testing capabilities for various countries in various periods. We also provided monthly probabilities of morbidity and fatality rates per 1 million population, depending on the number of tests performed in the countries around the world.

In this paper we propose that the number of SARS-CoV-2 tests be compared to the previous month’s diagnosed SARS-CoV-2 cases. This can easily be put into practice, i.e., decision makers can make future estimates based on historical (prior month) figures. The relations obtained have practical implications and can support planning of a SARS-CoV-2 testing process. We recommend increasing the ratio of monthly number of tests performed in the countries measured to the number of diagnosed in the prior month SARS-CoV-2 cases up to 12 or 43. We fully acknowledge that the higher the number of infections detected the more challenging for the country it is to increase the test numbers. Consequently, while countries may relatively easily follow our recommendations with a lower number of infections, they can become unable to follow them with larger ones. In such cases, we recommend considering a lockdown of selected branches of the economy.

The high dispersion of the data made us apply a probabilistic approach and calculate the probabilities of morbidity and fatality rates, depending on test numbers. Hence, once the results for medians of observations were obtained, we recalculated our results for the percentile with probabilities between 0.4 and 0.9. In the case of SARS-CoV-2 fatality rates (CFR) with a probability of 0.5, we calculated the following deaths numbers per 1 million population as follows: 50.9 (CTR 5); 24.2 (CTR 12); 15.7 (CTR 20); 11.1 (CTR 30); 8.2 (CTR 43); 7.2 (CTR 50); 5.4 (CTR 70) and 3.4 (CTR 120). As disclosed, despite a significant reduction in deaths up to a CFR of 43, a further (significant) increase of tests numbers results in only an insignificant reduction in the number of deaths. This is because the relationship identified in this paper between CFR and CTR follows a power law: CFR = 200/CTR^-0.85^. The relationship between CCR and CTR also follows a power law: CCR = 5900/CTR^-0.65^. The distributions of a wide variety of quantities follow a power law, including the sizes of activity patterns of neuronal populations, human judgments of stimulus intensity [35, 36] or the fluctuations of financial markets [37]. The universality of the power law is also applicable in our case.

The considerable differences in the distribution of SARS-CoV-2 cases, deaths, and tests performed have already been acknowledged by several scholars, who also provided selected reasons behind their findings [12]. The review of descriptive statistics undertaken at the beginning of this study endorses the hypothesis of these, i.e., the uneven distribution of SARS-CoV-2 cases, deaths, and tests among different countries. The probabilistic approach employed in this study makes the results obtained practical and applicable to various continents and countries, which can find themselves within different probability curves.

The development of the SARS-CoV-2 pandemic is frequently measured using the R ratio. This ratio indicates the average number of secondary cases produced by a primary case [13, 14]. Due to its simplicity and straightforward interpretation, this ratio has become popular among both researchers and decision makers. It should be noted that this ratio has already been employed in previous epidemics, in various regions [50, 51]. Scholars and decision makers are using the R ratio primarily to assess the effectiveness of non-pharmaceutical interventions in various countries over different periods [52]. Several scholars proposed employing the R ratio to assess what portion of the population requires to be vaccinated to reach herd immunity [16, 53]. The R ratio is also used to track real-time pandemic development [52, 54]. Despite the fact that the ratio clearly comments on epidemic development, it does not provide any evidence on the source of improvement or deterioration. This is because the R ratio is affected by multiple variables, which comprise, inter alia, control measures, contact rates, including lockdowns or bans on public events or climatic conditions [52, 55]. In this paper, we studied a specific variable, the number of SARS-CoV-2 tests. We selected this variable because decision makers have influence over it and identified that this variable has a significant influence on pandemic development. Here we explain how the monthly SARS-CoV-2 tests numbers relative to the prior month’s SARS-CoV-2 cases reported affect morbidity and fatality rates. Additionally, we have identified a strong and positive correlation between the absolute value of tests performed and the morbidities and fatalities. The latter indicates that the decision makers tend to increase the tests numbers in line with the pandemic development, rather as a prevention. The variables employed in this study, in contrast to the R ratio, do not comment on general epidemic development but inform how the number of Sars-COV-19 tests performed affects epidemic development. As a result, our findings are more practical for decision makers.

Finally, we shall note that this study has several limitations. Our sample does not comprise observations from all countries around the world. This is because not all countries provide such information publicly. However, we do not consider that these missing observations would significantly change our result. This study sample is already large and represents countries from every continent. Secondly, it should be stated that because a portion of SARS-CoV-2 patients undergo the infection with mild or even no symptoms, and because not all people with SARS-CoV-2 symptoms seek medical advice or testing, the ratios used in our study might not reflect all infected patients. The third limitation refers to the use of monthly observations in our study. This was done because of the lag between detection of new infections and deaths. With respect to the latter two limitations, we consider the values obtained, but not our results, could be subject to minor changes. As a result, we consider the power law approximation and probabilistic approach would remain applicable, with minor adjustments proposed to the approximated coefficients in the equations. Hence, our findings should still be sound and generalisable. Finally, we shall note, at the time our paper was written, vaccination had begun around the world. Despite the process of SARS-CoV-2 vaccinations being a complex and long-term one, i.e., would probably last over a year around the world, we consider the introduction of vaccinations might alter the pandemic development, and as a consequence the results provided in this paper, to some extent. Therefore, further studies on how the vaccination process has reduced SARS-CoV-2 morbidity and mortality rates and the ongoing global crises would be remarkably interesting.

## Conclusions

Accurate application of high volumes of SARS-CoV-2 diagnostic tests, if followed by the rapid use of the results to isolate and to apply appropriate therapy to infected individuals, could end or significantly limit the pandemic development. However, the financial and operational limitations of the testing capabilities of various countries do not allow for mass testing and force decision makers to look for optimum test levels. In this study, we have analysed the mutual relationships between the variable defined as the number of SARS-CoV-2 tests performed in a country in 1 month to the number of SARS-CoV-2 cases reported in a prior month and the probabilities of morbidities and mortalities per 1 million population. This probabilistic approach allows us to predict the morbidity and mortality rates in the following month based on the number of SARS-CoV-2 cases reported in a given month, assuming a different number of planned tests. We based our study on 1058 monthly observations relating to 107 countries, from six different continents in an 11-month period from March 2020 to January 2021. We used the moving percentiles for different probability values. We have found that the relationships between the morbidity and fatality rates and the test rate accurately describe the power law with exponents − 0.65 and − 0.85 respectively.

Next, we divided the relationships under investigation into three ranges according to ABC classification: most important, moderately important, and relatively unimportant for managing and control. The calculated Spearman rho and Kendall tau-b coefficients showed the importance of the correlations identified, and confirmed that the relationships between test numbers and morbidity and fatality rates are negative. We fitted each moving percentile by a three-segment piecewise-linear approximation with lines corresponding to the three identified ranges. The coefficients of these lines can be obtained by multiplying the coefficients of the Theil-Sen trend lines determined from observations in each range by the factor which depends only on the probability of a percentile. In the most important range A, corresponding to a small proportion of the number of SARS-CoV-2 tests performed in a month to the number of SARS-CoV-2 cases reported in a prior month, the slope of the approximating line is the largest and negative. The morbidity and fatality rates in this range are sensitive to any change in the number of tests. In the moderately important range B, the slope of the approximating line has a slope several times smaller, so increasing the number of tests in this range is much less effective. We recommend increasing the monthly test number up to the limit of this range i.e., to the point where the proportion of the number of tests performed in a month to the number of cases reported in a prior month is around 43. Further increase of test number is inefficient, because the slope of lines approximating percentiles is almost flat (range C).

We would like to note that the probabilistic approach used in this work can be applied e.g., to observations collected from only one country averaged over another period. The results obtained with our approach depend on the quality of data and they should be updated during the spread of the pandemic to obtain better predictions. Finally, we would like to mention that the demonstration in this paper of a power-law relation in SARS-CoV-2 data can help in understanding the mechanisms that underline the nature of epidemics.

## Data Availability

The datasets generated and/or analysed during the current study are available in the Our World in Data repository, https://ourworldindata.org/coronavirus-testing#our-checklist-for-SARS-CoV-2%20-testing-data. Further information on data and materials used are available from the corresponding author on reasonable request.

## Abbreviations

CCR: New SARS-CoV-2 cases reported in a month to 1 million population of the country
CFR: New SARS-CoV-2 deaths reported in a month to 1 million population of the country
CTR: Number of SARS-CoV-2 tests performed in a country in a month to the number of SARS-CoV-2 cases reported in a prior month
SARS-CoV-2: Severe acute respiratory syndrome coronavirus 2
